# Caffeine's Vascular Mechanisms of Action

**DOI:** 10.1155/2010/834060

**Published:** 2010-08-25

**Authors:** Darío Echeverri, Félix R. Montes, Mariana Cabrera, Angélica Galán, Angélica Prieto

**Affiliations:** Laboratorio de Investigación en Función Vascular, Departamento de Investigaciones, Fundación CardioInfantil—Instituto de Cardiología, Carrera 13b no. 163-85, Torre A, Piso 3., Bogotá, Colombia

## Abstract

Caffeine is the most widely consumed stimulating substance in the world. It is found in coffee, tea, soft drinks, chocolate, and many medications. Caffeine is a xanthine with various effects and mechanisms of action in vascular tissue. In endothelial cells, it increases intracellular calcium stimulating the production of nitric oxide through the expression of the endothelial nitric oxide synthase enzyme. Nitric oxide is diffused to the vascular smooth muscle cell to produce vasodilation. In vascular smooth muscle cells its effect is predominantly a competitive inhibition of phosphodiesterase, producing an accumulation of cAMP and vasodilation. In addition, it blocks the adenosine receptors present in the vascular tissue to produce vasoconstriction. In this paper the main mechanisms of action of caffeine on the vascular tissue are described, in which it is shown that caffeine has some cardiovascular properties and effects which could be considered beneficial.

## 1. Introduction

Coffee is one of the most frequently consumed beverages in the world. It represents a culture and an economy. It has been produced in Colombia since the nineteenth century and is the main export to more than 36 countries; in 2008 it represented close to 12.4% of the harvest of mild Arabica type coffee and 12.2% of worldwide coffee exports. There are approximately 590 coffee growing municipalities, 513,000 coffee growers, 640,000 direct employees, and more than a million indirect employees, which means approximately 2 million people dependent on coffee cultivation [1] 

Over 2,000 substances have been isolated from coffee. The major component of coffee is carbohydrates, which make up 38–42% of the toasted coffee bean, followed by lipids and amino acids with about 20 and 10%, respectively. Melanoidins make up 23% of the weight and are what give the beans their brown color. They also contain minerals, aliphatic and chlorogenic acids, trigonellines, and volatile aromas. Of the alkaloids, the most studied and recognized one is caffeine, which makes up 1.3 to 2.4% of the bean's weight [2] followed by other purinic alkaloids such as theobromine and theophylline and pyridine such as trigonelline. 

Coffee consumption is generally associated with a large number of diseases and health alterations. However, the majority of epidemiological studies regarding this relationship have not yielded a clear conclusion, mainly due to the lack of concrete and continuous information regarding the frequency of consumption, the exact composition of the beverage, and factors associated with an unhealthy lifestyle (cigarette smoking, alcohol, and sedentarism). These aspects in combination could lead to diseases or health problems [3]. 

Many epidemiologic studies have studied the relationship between coffee consumption and the risk of heart disease. An analysis [4] of the coffee-mortality relationship states that there is no direct relationship between coffee consumption and an increase in mortality; on the contrary, the authors describe a slightly Inverse relationship between the consumption of coffee and their benefits related to the inflammatory process, endothelial function, and the risk of developing type 2 diabetes. According to Yukawa et al. [5] the regular consumption of coffee reduced susceptibility to low-density lipoprotein oxidation, a pathway which develops in atherosclerotic plaques, thus favoring endothelial function. In another vein, it has been shown that some coffee components, especially phenolics (chlorogenic acid, ferulic acid), have a great antioxidant capacity [6], and the consumption of coffee is associated with a small reduction in mortality in women with hepatic disease and/or cirrhosis and shows a protective effect on the liver in hepatic cancer [7]. Additionally, caffeine increases the production of urine with water and electrolyte secretion patterns very similar to those seen with the thiazides [8]. The underlying mechanisms may depend on various factors such as dose, chronic exposure, genetic and enzymatic factors, among others. In animal studies with caffeine exposure, an increase in glomerular filtration and kidney blood flow, especially in the renal medulla, is seen. In a study of the intrarenal mechanisms responsible for the natriuretic effect of caffeine, the renal secretion of sodium increased, and the glomerular filtration rate remained the same, suggesting that diminished fractional sodium reabsorption, both in the proximal and distal tubule of the nephron, contributes to the natriuretic effect of caffeine [8] 

Caffeine is the psychoactive substance most widely consumed in the world, it is found not only in coffee but also in tea, carbonated beverages or soft drinks, chocolate, and a wide variety of medications, including appetite suppressants, diuretics, analgesics, and decongestants; the majority of which are sold over the counter and do not have a regulatory control [9, 10]. If you combine the consumption of coffee, tea, chocolate, and soft drinks, the general population consumes a considerable amount of caffeine per day. Adults over the age of 25 have an estimated consumption of approximately 2.4 mg/kg/day, while children under the age of 12 have an estimated consumption of approximately 0.7 mg/kg/day. In addition, it has been confirmed that theobromine and theophylline are alkaloids also found naturally in green tea, black coffee, and cacao [11] however, the direct effect of these substances on physiological responses to the ingestion of foods and beverages containing these types of alkaloids, and the role of each, is not clear.

## 2. Metabolic Pathway of Caffeine and Its Metabolites

Caffeine is metabolized into more than 25 metabolites in humans, mainly Paraxanthine, Theobromine, and Theophylline [12] 

Caffeine metabolism yields paraxanthine as a final product, which represents 72 to 80% of caffeine metabolism. There are five main metabolic pathways which contribute to caffeine metabolism in adults [13, 14]. The first three consist of demethylization of N-3 to form Paraxanthine, N-1 to form Theophylline (vasodilator, increased cerebral and muscular blood flow), and N-7 to form Theophylline (vascular, bronchiole, muscular, and respiratory relaxant). The hepatic cytochrome P-450 (CYP) isoenzyme metabolizes most of the caffeine (95%) by three demethylizations which on average give an in vivo metabolism percentage of 85% paraxanthine, 10% theobromine, and 5% theophylline [15]. The fourth pathway results in the formation of uracil metabolites, and the fifth consists of renal elimination of the remaining percentage of caffeine that was not able to be degraded in the process. 

The large interindividual differences observed in plasmatic concentration of caffeine following the administration of an equal dose are mainly due to variations in metabolism. These variations depend on four factors: genetic polymorphisms, metabolic induction and inhibition of cytochrome P-450, individual (weight, sex), and the presence of hepatic diseases [14] 

Caffeine is absorbed rapidly and completely from the intestinal tract, making it 100% bioavailable. The time in which maximum plasmatic concentration is obtained (Tmax) is 30–45 minutes [11, 14, 16, 17] fasting and is delayed with food ingestion; it has an average metabolic half life in humans of 2.5 to 4.5 hours [18]

## 3. Vascular Effects of Caffeine

Numerous studies have been carried out to determine the effect of caffeine on the cardiovascular system, with inconclusive results. Some have found that the consumption of caffeine increases cardiovascular risk [19–21] while others have described a beneficial or neutral effect on the same [22–24]. It is evident that the cardiovascular response to this substance depends on a variety of factors such as the amount ingested, the time of consumption, the frequency, degree of absorption, and hepatic metabolism, all aspects which cause a unique response of each individual to caffeine [25]. In addition to these factors, it is believed that some substances found in caffeinated beverages (theobromine and theophylline active substances in bronchodilator medications used in the treatment of respiratory diseases) could have some effects on the variability of these particular physiologic responses. 

Caffeine is a xanthine which acts in the body's cells by different mechanisms of action and on a wide range of molecular targets. It intervenes as an antagonist of the adenosine receptors, inhibitor of phosphodiesterase enzymes, sensitizer of calcium liberation channels, and GABA receptor antagonist [26]. Other cardiovascular processes are related to the reduction of cytoplasmic Ca^2+^ in the vascular smooth muscle cell (VSMC) through cyclic adenosine monophosphate (cAMP) and the increase of the same in the endothelial cell, favoring the synthesis of nitric oxide (NO). 

We know that other related substances with a similar structure to that of the alkaloids present in coffee currently provide an important research tool towards the development of potential treatments for Alzheimer disease, asthma, cancer, diabetes, and Parkinson's disease [14]. In this paper, the main mechanisms of action of caffeine on the vascular tissue are described, and we will try to break a series of myths and paradigms that have negatively influenced the consumption of coffee. These mechanisms are summarized in Table 1.

## 4. Mechanisms of Action of Caffeine at the Endothelial Level

The endothelium is probably the most extensive tissue in the human body. It forms an anatomic and functional barrier covering the arterial walls which is highly selective and permeable through a continuous, uninterrupted, and soft surface. It synthesizes and releases a broad spectrum of vasoactive substances, intervening in the regulation of VSMC tone through an interaction between vasoconstrictor (renin, angiotensin, ET-1, etc.) and vasodilator substances (NO, PgI2, endothelium-derived hyperpolarizing factor, bradykinin, etc.) [45, 46]. 

Caffeine acts directly on the endothelial cell, stimulating the production of NO [40]. This effect was evaluated by blocking the NO pathway with NG-nitro-L-arginine, oxyhemoglobin, and methylene blue [47]. NO is synthesized by nitric oxide synthase (eNOS) from L-arginine and oxygen. In order for it to be produced, calmodulin must be bound to the enzyme, and it only binds in the presence of Ca^2+^, which it obtains from the cytoplasmic content [48]. 

In the endothelial endoplasmic reticulum, the ryanodine receptor activity is stimulated by caffeine, concentrations of Ca^2+^, and adenine nucleotides. Caffeine stimulates the release of Ca^2+^ from the reticulum, increasing its concentration in the cytoplasm (iCa^2+^), forming a complex with calmodulin which favors the activation of eNOS. This mechanism is compatible with the general characteristics of the *calcium-induced calcium release *(CICR) [27, 29, 49], in which a minimal quantity of Ca^2+^ is required in the cytoplasm: not enough to activate the eNOS but enough to stimulate the release of more Ca^2+^ from the reticulum, increasing the iCa^2+^. It seems that caffeine lowers the threshold for the activation of CICR, which means that the mechanism is activated with practically at rest Ca^2+^levels [28]. In VSMC, the entrance mechanisms of Ca^2+^ responsible for a sustained cellular activation are normally mediated both by voltage-operated Ca^2+^ channels as well as specific receptor [50]. 

To summarize, the effect of caffeine on the vascular endothelium is a greater expression of NO [21], which has an autocrine effect, acting on the same endothelial cell to increase Ca^2+^, potentiating the response, and coming out of the endothelial cell to diffuse rapidly to the VSMC in a paracrine fashion [51]. 

**Figure 1:**
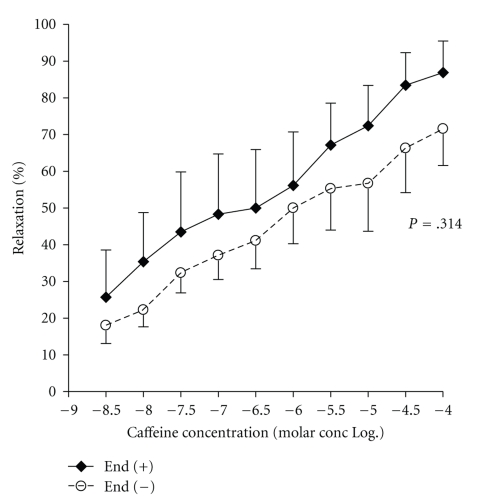
Relaxation of human arteries in the presence of increasing doses of caffeine. End (+): normal endothelial function; End (−): endothelial dysfunction. The data are presented as average ± standard error of the media, which is presented in only one direction to facilitate the reading of the figure. It is reproduced with the authorization of Biomédica.

Some authors argue that caffeine produces greater vasodilation by acting on the endothelium than on the VSMC [33]. However, in *in vitro *studies carried out by our group, using rabbit arteries[52] and human internal mammary arteries, we observed that caffeine induces a potent arterial vasodilator effect in the presence or absence of preserved endothelial function (Figure 1).

## 5. Caffeine Mechanisms of Action on Smooth Muscle Cells

Caffeine may exert vascular mechanisms of action through its direct or indirect effect on the VSMC. 

### 5.1. Direct Effects

Caffeine, by acting on the VSMC, generates a minimal initial contraction and then a significant vasodilator effect. There are various mechanisms that explain these effects. 

#### 5.1.1. Caffeine and the Ryanodine Channels

The direct action of caffeine on the VSMC occurs initially through the ryanodine channels of the sarcoplasmic reticulum, stimulating the CICR mechanism, which generates an increase in iCa^2+^ and a slight transitory contraction [22]. This response is independent of the amount of extracellular Ca^2+^ and the presence of Ca^2+^ channel blockers [53]. 

**Figure 2:**
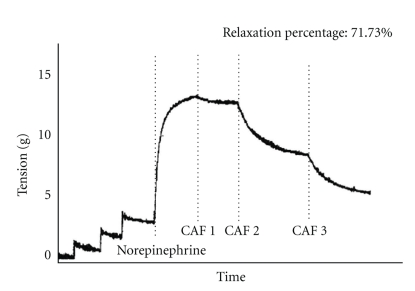
Typical *in vitro* vascular response curve of caffeine produced in rabbit aortas with three accumulated caffeine doses (corresponding to the plasmatic concentration obtained upon consumption of one, two, and three espressos). Vasodilation induced by the administration of caffeine without prior contraction is shown. It is reproduced with the authorization of Revista Colombiana de Cardiología

As the intrareticular Ca^2+^ is used up, the entrance of extracellular Ca^2+^ to the cell through the slow (L-type) channels and the nonselective cation channel in the cell membrane begins. Caffeine directly activates the nonselective cation channel [30] to increase iCa^2+^. This increase in iCa^2+^ prolongs the contraction started by the CICR. It is interesting to note that in the experiments carried out with caffeine in our laboratory [54], in human arteries and animal models, this contraction was not seen, which leads us to believe that it is probably a very slight vasoconstrictor effect (Figure 2).

#### 5.1.2. Caffeine and cAMP

*In vitro *experiments carried out with caffeine have demonstrated that in spite of an increase in the VSMC iCa^2+^, a vasodilator effect is seen [55, 56]. Caffeine is a nonselective competitive inhibitor of the phosphodiesterase enzymes [40]. These enzymes have the capacity to degrade the phosphodiesterase bond in some compounds such as cAMP and cyclic guanosine monophosphate (cGMP). One of the main enzymes inhibited by caffeine is 3′-5′ AMP phosphodiesterase [31, 32], whose function is to degrade cAMP, causing its local accumulation. The antiphosphodiesterase activity is concentration dependent, inhibiting the enzyme up to 5% at concentrations of 1×10^−4^ M and up to 80% at concentrations of 1×10^−2^ M [29]. In addition, it is time dependent, generating a greater accumulation of cAMP the longer the incubation time [28]. 

The accumulation of cAMP generates an increase in the phosphorylation of the kinase enzyme of the myosin light chain (MLC) in the cell's contractile apparatus (actin-myosin). In this state, the enzyme is less sensitive to Ca^2+^, and therefore its activity is diminished. As the enzyme is inhibited, the MLC phosphorylation is diminished and the actin-myosin interaction is inhibited. This results in an increase of intracellular Ca^2+^ concentration without contraction^32^, which has been described as a loss of “sensitivity” to Ca^2+^ [28, 57]. As MLC phosphorylation decreases, the activity of MLC-phosphatase and relaxation predominate. 

Up until now, the kinase enzyme of the myosin light chain in smooth muscle is the enzyme that activates the MLC through phosphorylation to a specific domain. The agonist stimulation increases the intracellular concentration of Ca^2+^ in smooth muscle, causing it to bind to calmodulin, which when bound to Ca^2+^ activates the kinase enzyme in the myosin light chain, thereby activating the form that interacts with actin to cause contraction. However, more recent studies have shown that this mechanism is not the only regulator of the myosin-actin interaction [58]. 

Rembold et al. [36] observed that upon adding 20 mM of caffeine to precontracted arteries, there was an increase in iCa^2+^ without a significant increase in tone, which could not be explained solely by the increase in phosphorylation of MLC kinase. They documented that the Ca^2+^ had a heterogeneous distribution. They concluded that caffeine increases iCa^2+^ but in a region distant from the contractile apparatus, which therefore did not result in a contraction. It is probable that this effect of caffeine is mediated by cAMP, since cAMP also increases the “non-contractile” Ca^2+^ [59]. 

However, the effects of caffeine described cannot be attributed solely to the increase in cAMP. In 1990, Ozaki et al. [56] carried out an observation of precontracted arteries, to which caffeine or forskolin (which also increases cAMP) were added. At similar levels of cAMP in the two preparations, caffeine inhibited contraction of the VSMC to a greater degree than forskolin.

#### 5.1.3. Other Direct Mechanisms

 Caffeine also inhibits inositol triphosphate (IP3) compound which stimulates the secretion of Ca^2+^ from the sarcoplasmic reticulum and is indispensable for contraction. This inhibitory effect of the IP3 pathway by caffeine is antagonized by the addition of ATP [34]. Given that the xanthenes contain an adenine ring identical to that of ATP, it has been postulated that they can interact competitively with the ATP binding site on the IP3 receptor [60]. In addition, caffeine acts directly on the voltage-dependent Ca^2+^ channels in the plasmatic membrane to inhibit the entrance of Ca^2+^ [37], an effect which is independent of its antiphosphodiesterase action [38]. 

**Figure 3:**
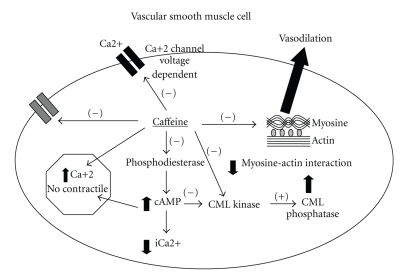
Vasodilation produced by the direct effects of caffeine on the VSMC. Caffeine inhibits the voltage-dependent Ca^2+^ channels and the entrance of calcium to the cytoplasm. In addition, it inhibits the IP3 receptor and increases the “non-contractile Ca^2+^. Due to its antiphosphodiesterase action, there is an accumulation of cAMP, which increases the non-contractile Ca^2+^, diminishes cytoplasmic Ca^2+^ (iCa^2+^), and inhibits Myosin Light Chain Kinase (MLC Kinase). Therefore MLC phosphatase predominates and there is vasodilation. Caffeine also directly inhibits MLC Kinase and the actin-myosin interaction.

Ozaki et al. [35] also demonstrated that caffeine acted directly on the MLC kinase and on the actin and myosin interaction, slightly inhibiting MLC phosphorylation and contraction. The direct mechanisms of vasodilation are illustrated in Figure 3. 

More recently, Sandow et al. [61] stated that the modulation of vascular cellular calcium (control of vascular tone, flow, and blood pressure) is regulated by specialized signaling microdominions in vascular smooth muscle cells, spatially located in Ca^2+^ channels and receptors, and interacting functionally; some studies suggest that these sites are also present in endothelial cells.

### 5.2. Indirect Effects

**Figure 4:**
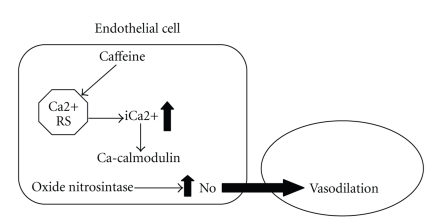
Indirect effects of caffeine on VSMC. Caffeine acts on the endothelial cell increasing cytoplasmic Ca^2+^ which will form the calcium-calmodulin complex which activates the nitric oxide synthase enzyme to produce nitric oxide. This diffuses to the VSMC.

The indirect effects of caffeine on the VSMC occur through NO, synthesized by the eNOS in the endothelial cell, which diffuses rapidly to the VSMC. These effects are illustrated in Figure 4. 

As NO enters the VSMC, it binds to the heme group of the guanylate cyclase enzyme, activating it. This catalyzes the conversion of GTP to cGMP, which increases the activity of a series of cGMP-dependent protein kinases (PKCs), particularly the I*α* type [62]. The PKI*α* stimulates the dephosphorylation of the MLC through phosphatase, producing vasodilation. The PKCs and cGMP also diminish cytoplasmic Ca^2+^ and inhibit IP3 [30]. Caffeine, in turn, competitively inhibits 3′5′ cGMP phosphodiesterase [20], stimulating even more accumulation of cGMP.

## 6. Other Mechanisms of Action

### 6.1. Action through Adenosine Receptors

There are different types of adenosine receptors labelled A1, A2a, A2b, and A3. Caffeine acts as a competitive inhibitor of the A1, A2a, and b receptors [63]. Caffeine competitively blocks these receptors as demonstrated in the experiment carried out by Sattin and Rall in 1970 [39], but this effect was reversed if more ATP (adenosine precursor) was added to the preparation. Paraxanthine, which is the main metabolite of caffeine, is an even more powerful blocker of these receptors than caffeine [2]. 

The action of adenosine depends on the type of receptor it stimulates and the type of tissue or cell in which it is found. The direct effects of adenosine on the different vascular systems are summarized in Table 2. The local vascular effects of adenosine are primarily vasodilation of the different beds. This effect depends mainly on the A2a receptors which are found in high concentrations in vascular tissue [57]. 

Caffeine, by competitively blocking the adenosine receptors, increases its plasmatic concentration [64] which increases its systemic effects. At a systemic level, adenosine stimulates the chemoreceptor distributed throughout the circulation, causing a generalized increase in sympathetic tone, with an increase in circulating catecholamines, peripheral vascular resistance, and renin secretion [44, 65]. Several studies have documented an increase in systolic arterial pressure of 6 to 7.5 mmHg and 2.6 to 4 mmHg in diastolic pressure 60 minutes after the administration of 300 mg of caffeine (equivalent to drinking a triple espresso) [18, 43]. 

In spite of this “indirect” vasoconstrictor effect produced by caffeine, it is important to point out that the chronic consumption of caffeine creates a tolerance to its adenosine receptor-dependent effects. Chronic blocking of the adenosine receptors, inducing “*upregulation*” (an increase in the number and sensitivity) of the receptors has been described with a low-moderate caffeine consumption (approximately two cups of coffee for more than 5 days) [66]. A meta-analysis carried out in 1999 [67] described an increase in the systolic and diastolic arterial pressure (2.4 and 1.2 mmHg, resp.) with the chronic consumption of 5 cups of coffee a day, on average, which is a considerably lower value from that obtained in studies carried out on subjects who are not caffeine consumers.

This “*up-regulation*” generates the “abstinence syndrome” described by Griffiths in 1988 [68], characterized by headache, fatigue, flushing, and anxiety. When you abruptly stop the consumption of caffeine in a habitual consumer, there is a greater number of available adenosine receptors, which potentiates the vasodilation produced by adenosine, causing the symptoms [59, 69, 70]. 

It has been asserted that the predominant cardiovascular effects of caffeine occur at the adenosine receptors because much lower concentrations are required (*μ*m) than those used in studies that show their effect on Ca^2+^ and phosphodiesterase (mM), which are concentrations that are not attained *in vivo *[71]. However, in our *in vitro *studies which were carried out with micromolar (*μ*m) concentrations of caffeine there was a significant vasodilator effect (approximately 75%) at human consumption concentrations [46]. *In vitro *studies do not evaluate the systemic response to caffeine, and therefore it is not clear yet which one of the mechanisms of action predominates *in vivo*, given that there are various factors that affect its metabolism and its effects. 

#### 6.1.1. Caffeine in Relation to Migraine Type Headaches

Migraines are irregular and episodic which is why there is no specific explanation for why a migraine occurs at any given time. In general it is supposed that exposure to certain environmental factors combined with individual internal factors causes migraine episodes. There are reports that certain dietary, physical, hormonal, emotional, and environmental factors trigger or cause migraine episodes. Those most frequently reported include stress, alcohol, foods, excess or lack of sleep, and weather conditions. 

Headaches (migraine) may be related to caffeine consumption due to its removal from the usual diet, causing an abstinence syndrome: an alteration in the normal functioning of the nervous system. The mechanism by which this occurs is a blocking of the adenosine receptors; when there is an excessive release of adenosine there is a response in which the release of neurotransmitter molecules, such as serotonin, noradrenaline, acetylcholine, and dopamine, is inhibited, causing an imbalance that can be seen in the symptoms associated with migraines [72] 

There is no clear conclusion that migraines can be caused by caffeine. Adenosine has opposite effects depending on its site of action; centrally, in the brain and spinal cord, adenosine acts as an analgesic, but peripherally it can cause pain. Adenosine dilates blood vessels in the head and neck. The concentration of adenosine in the head and neck increases approximately 68% above normal concentrations during migraine episodes, causing vasodilation and pain [73].

The nervous system compensates the interference of caffeine by releasing more adenosine, increasing the number of adenosine receptors in the neuron surface, increasing the affinity of these receptors and decreasing the rate at which adenosine molecules are removed. All these changes tend to increase the activation of adenosine receptors, to compensate the receptors occupied by caffeine.

Caffeine is also a common ingredient in many medications used for treating migraines, due to the fact that it makes analgesics work more efficiently, causes a faster absorption, and allows for a reduced dosage which decreases possible side effects of certain analgesics.

### 6.2. Action through the Activation of the Autonomic Nervous System

Caffeine, as it blocks the adenosine receptors, stimulates a reflex activation of the sympathetic system in conscious patients. Corti et al. [43] demonstrated that in habitual coffee consumers the sympathetic system is activated, but this does not lead to a significant increase in the peripheral vascular resistance, while in nonconsumers, coffee stimulated the sympathetic system and increased arterial pressure. In this study it was shown that the consumption of coffee produced an increase in sympathetic tone after ingesting regular and decaf coffee. Although several studies attribute the increase in arterial pressure to caffeine [61, 74], it is possible that there are other substances present in coffee involved in the increase in sympathetic tone and arterial pressure. In addition, it is important to differentiate that the results of different studies regarding the effect of caffeine on arterial pressure show a variation according to the population group (hypertensives, stress factors, and age) and also of the design and purpose of each one of these studies. According to this analysis, the most accurate conclusion is that tolerance developed with the regular consumption of caffeine diminishes the effect of the same on arterial pressure approximately 30 minutes after ingestion, with an increase peak in the range of 1 to 2 hours and a persistence of approximately 4 hours [75]. 

Studies have shown that caffeine increases plasmatic levels of stress hormones, including catecholamines such as adrenaline and noradrenaline and cortisol. These humoral effects indicate that both the sympathetic-adrenal medullary system as well as the adrenocorticoid components of the neuroendocrine response to stress are activated [76, 77].

The ingestion of caffeine suggests an increase in sympathetic nervous activity as well as a slight change in physiologic variables such as body temperature, blood pressure, and heart rate. It has been shown that many pharmacologic effects of caffeine are related to the sympathetic nervous system. Certain doses, especially high ones, may cause tachycardia, a significant increase in plasmatic adrenaline concentrations, an increase in the plasmatic activity of renin, as well as thermogenic and lipolytic effects. This effect on sympathetic activity presents variable results and continues to be controversial and only partially understood [78]

### 6.3. Action through the Renin-Angiotensin-Aldosterone Axis (RAA)

Caffeine has three main effects on the RAA axis [41]. First of all, it blocks the inhibiting effect of adenosine on the juxtaglomerular cells in the kidney, increasing the secretion of renin [79]. In addition, due to its antiphosphodiesterase activity, it increases the concentration of cAMP, which is a precursor of rennin, and also increases the secretion of renin by activating the sympathetic system [42]. Theoretically, this increase in renin secretion results in vasoconstriction and an increase in peripheral vascular resistance. 

Caffeine only has this effect in conditions in which renin is elevated (e.g., cirrosis, congestive heart failure) and not in normal physiologic conditions. For this reason, in healthy people, caffeine does not significantly affect renin production [80, 81].

## 7. Conclusions

Coffee is one of the most consumed beverages worldwide and is the primary export of Colombia. It has a composition of over 2,000 substances, with a predominance of carbohydrates, lipids, amino acids, melanoidins, and the most important and well-known of all caffeine. In this paper some of the currently known vascular mechanisms of action of caffeine are described. 

Caffeine is a xanthine which displays several mechanisms of action on the vascular wall, especially on the endothelial tissue and the vascular smooth muscle cell VSMC. At the same time, it is known that it acts on the autonomic nervous system and on arterial pressure, with a possible development of tolerance with regular consumption. 

The effects it produces are the result of the activation or blocking of different types of receptors, such as those of adenosine, IP3, NO, among others. In addition, its effects seem to be contradictory depending on the cellular structure and the time of exposure over which it acts. A mild and transitory vasoconstrictor effect exists, which depends mainly on the caffeine concentration in the VSMC. However, the main and predominant effect of caffeine on the vascular wall is vasodilating, acting equally on the VSMC directly or indirectly and also on the endothelial structure. At the endothelial level, nitric oxide is liberated and as a result produces arterial vasodilation. It has been shown that this effect is caused in the presence or absence of preserved endothelial function. 

As for the effects on the vascular smooth muscle cell, caffeine causes direct and indirect effects according to the type of stimulus, either at the level of cellular Ca^2+^ concentrations or on competitive effects with specific enzymes. Indirectly, the diffusion of nitric oxide from the endothelial tissue towards the VSMC increases the vasodilator effect. 

In spite of being a widely consumed substance worldwide, its vascular effect, and cardiovascular effect in general, continues to be controversial. It is evident that the effects of coffee consumption vary notably according to the population being studied and specific metabolic and pathologic factors. For this reason, it is necessary to continue the search for greater information regarding the effects and mechanisms of action of caffeine, in order to determine the impact of the mechanisms as risk factors or if said mechanisms can be considered protective at a cardiovascular level.

## Figures and Tables

**Table 1:** Summary of the vascular effects of caffeine.

Structure	Type of effect	Effect	Reference
Endothelium	Direct	Activates the ryanodine channels in the ER	Zucchi,1997 [27]
			Endo, 1977 [28]

VSMC	Direct	Activates the ryanodine channels in the ER	Karaki, 1988 [29]
		Activates the nonselective channel for cations	Guerrero, 1994 [30]
		Inhibits the cAMP phosphodiesterase	Butcher, 1963 [31]; Ahn, 1988 [32]
			Hatano, 1995 [33]
		Inhibits the IP3 receptor	Missiaen, 1994 [34]
		Inhibits MLC kniase	Ozaki, 1990 [35]
		Increases “noncontractile” Ca^2+^	Rembold, 1995 [36]
		Inhibits voltage-dependent Ca^2+^ channels	Martin, 1989 [37]; Hughes, 1990 [38]
		Blocks adenosine receptors	Sattin, 1970 [39]

VSMC	Indirect	Increases the production of nitric oxide	Hatano, 1995 [33]; Umemura, 2006 [40]
		Increases the production of renin	Tofovic, 1996 [41]; Jackson, 1991 [42]
		Stimulates the sympathetic system	Corti, 2002 [43]; Robertson, 1978 [44]

VSMC: vascular smooth muscle cell, ER: endoplasmic reticulum, Ca^2+^: calcium, and MLC: myosin light chain.

**Table 2:** Vascular effects of adenosine.

Vasculature	Effect	Receptor
Coronary	Vasodilation	A2a

Pulmonary		
1. Pulmonary artery	Vasoconstriction	A1
	Vasodilation	A2a
2. Microcirculation	Vasodilation	A2b

Mesenteric	Vasodilation	Unknown

Renal		
1.General circulation	Vasodilation	A2a
2. Afferent arteriole	Vasoconstriction	A1

Aorta	Vasodilation	A2b

## Abbreviations and Acronyms

cAMP: cyclic adenosine monophosphate AMPc
ATP: Adenosine triphosphate
CICR: Calcium induced calcium release
MLC: Myosin light chain
VSMC: Vascular smooth muscle cell
eNOS: Endothelial nitric oxide synthase
ET-1: Endothelin-1
cGMP: Cyclic guanosine monophosphate
IP3: Inositol triphosphate
NO: Nitric oxide
PgI2: Prostaglandin I_2_.
