# Corrigendum to “Caffeine's Vascular Mechanisms of Action”

**DOI:** 10.1155/2019/7480780

**Published:** 2019-11-20

**Authors:** Darío Echeverri, Félix R. Montes, Mariana Cabrera, Angélica Galán, Angélica Prieto

**Affiliations:** Laboratorio de Investigación en Función Vascular, Departamento de Investigaciones, Fundación CardioInfantil—Instituto de Cardiología, Carrera 13b no. 163-85, Torre A, Piso 3., Bogotá, Colombia

In the article titled “Caffeine's Vascular Mechanisms of Action” [1], there was an error in section “2. Metabolic Pathway of Caffeine and Its Metabolites,” where it refers to three metabolites: Paraxanthine, Theobromine, and Theophylline. However, in the second paragraph the three were actually mentioned as Paraxanthine and Theophylline twice as follows:

“Caffeine metabolism yields paraxanthine as a final product, which represents 72 to 80% of caffeine metabolism. There are five main metabolic pathways which contribute to caffeine metabolism in adults [13, 14]. The first three consist of demethylization of N-3 to form Paraxanthine, N-1 to form Theophylline (vasodilator, increased cerebral and muscular blood flow), and N-7 to form Theophylline (vascular, bronchiole, muscular, and respiratory relaxant).”

Accordingly, the paragraph should read as follows:

“Caffeine metabolism yields paraxanthine as a final product, which represents 72 to 80% of caffeine metabolism. There are five main metabolic pathways which contribute to caffeine metabolism in adults [13, 14]. The first three consist of demethylization of N-3 to form Paraxanthine, N-1 to form Theobromine (vasodilator, increased cerebral and muscular blood flow), and N-7 to form Theophylline (vascular, bronchiole, muscular, and respiratory relaxant).”
